# Peripheral T-cell lymphoma presenting as testicular mass; a diagnostic challenge

**DOI:** 10.1186/1477-7819-11-68

**Published:** 2013-03-14

**Authors:** Saroona Haroon, Arsalan Ahmed

**Affiliations:** 1 Histopathology section, Department of Pathology and Microbiology, The Aga Khan University Hospital, Stadium road, Karachi, 78400, Pakistan

**Keywords:** T-cell lymphoma, Orchidectomy, Immunohistochemical, Histopathological

## Abstract

**Background:**

T-cell lymphomas involve the testis infrequently, which deserve special attention because of the poor prognosis and the need to make an appropriate diagnosis, which could lead to a better therapeutic strategy.

**Case presentation:**

A 40-year-old man presented with right testicular swelling for past three months. The swelling was painless, hard and rubbery. Testicular ultrasound showed diffuse increase in size of the testicle, with alteration in its echogenicity. The patient underwent orchidectomy, and based on histopathological and immunohistochemical tests, a peripheral T-cell lymphoma, not otherwise specified was diagnosed.

**Conclusion:**

Testicular peripheral T-cell lymphomas are rare and aggressive cancers, with clinical differentials of seminoma and non-neoplastic conditions.

## Background

Testicular lymphomas comprise 5% of testicular malignancies and these account for about 1% of all lymphomas. Most of these present at the age of over 60 years, with mean age 56 years. The testicular lymphomas are usually disseminated at the time of presentation [1,2]. The phenotype of the lymphomas is almost always B-cell type with most of the tumors being the diffuse large B-cell subtype; however, rarely, anaplastic lymphoma, Burkitt’s lymphoma, or Hodgkin’s lymphoma may involve the testis as the primary site [1-3]. T-cell lymphoma of the testis is rare whether as a primary or secondary tumor. Better prognosis is predicted if the tumor is a primary, localized to the testis (5-year survival 60% vs. 17% for disseminated disease/other stages) and unilateral. Best treatment options are orchiectomy and chemotherapy, along with radiation [4].

The gross morphology of a lymphoma appears as white-tan-pink coloured, and fleshy, resembling a seminoma, and it often presents with extratesticular involvement [2,4]. Microscopically splayed apart, but relative sparing of tubules by lymphoma cells is seen and foci of vascular invasion in are seen in 60% of patients. There is significant sclerosis in 30% of cases. The non-cohesive tumor cells have large irregular nuclei, and prominent nucleoli; the cells show moderate to marked pleomorphism. No intratubular germ cell neoplasia is seen, which is present in seminoma, a major differential diagnosis on morphology [2,4].

## Case presentation

A 40-year old Asian male patient reported to the general surgical outpatient department at Mayo Hospital, Lahore in October 2011 with the history of a rapidly enlarging painless lump in his right scrotum for the past two months. There were no comorbid conditions. He was married with three children; all of them were alive and healthy. His previous medical and surgical history was unremarkable.

On physical examination, a non-tender lump measuring 4.0 × 3.5 cm, firm-to-hard in consistency, was present as a right testicular swelling. It was associated with slight skin puckering. Examination of the other, that is, the left testis was unremarkable. There was no generalized lymphadenopathy on general physical examination. The liver and spleen were of normal size. Sonographic examination of the scrotum revealed a well-defined, hypoechoic space-occupying lesion in the cranial and lateral aspect of the right testis. Findings on sonographic and computerized tomography (CT) examinations of the abdomen were normal. Differential diagnosis for this sonographic appearance was seminoma and orchitis. His erythrocyte sedimentation rate (ESR) was within normal range and the Montoux test for *Mycobacterium tuberculosis* was negative. His complete blood counts at the time of presentation were within normal limits. On discussion with the patient, he was informed that benign pathology such as isolated tuberculous orchitis was a possibility and informed consent was taken.

**Figure 1:**
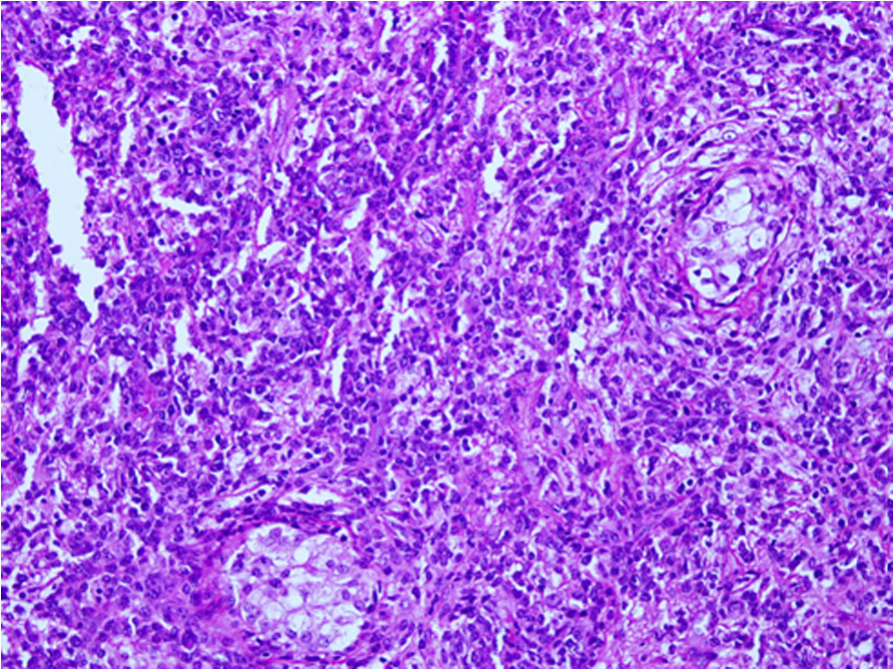
The tumor cells are splaying the seminiferous tubules apart and present in form of discohesive cells (Hemotoxylin and eosin, 20×).

Testicular biopsy was performed under general anesthesia and the specimen was sent in formalin to the Histopathology Laboratory, Aga Khan University Hospital, Karachi. The histopathological examination revealed markedly necrotic tissue with foci of the lesion. Histologic examination of the specimens revealed a diffuse proliferation of malignant round cells within the interstitium of the testicular parenchyma. The cells were present in the form of sheets with scant cytoplasm and slight variation in size and shape of medium-to-large nuclei, which frequently showed irregular foldings and granular chromatin. Mitotic activity was also observed with mitoses seen at a rate of 16/10 high power fields. Intervening scattered vessels were also present. These cells infiltrated around the seminiferous and epididymal tubules and presented in a discohesive pattern (Figure 1).

**Figure 2:**
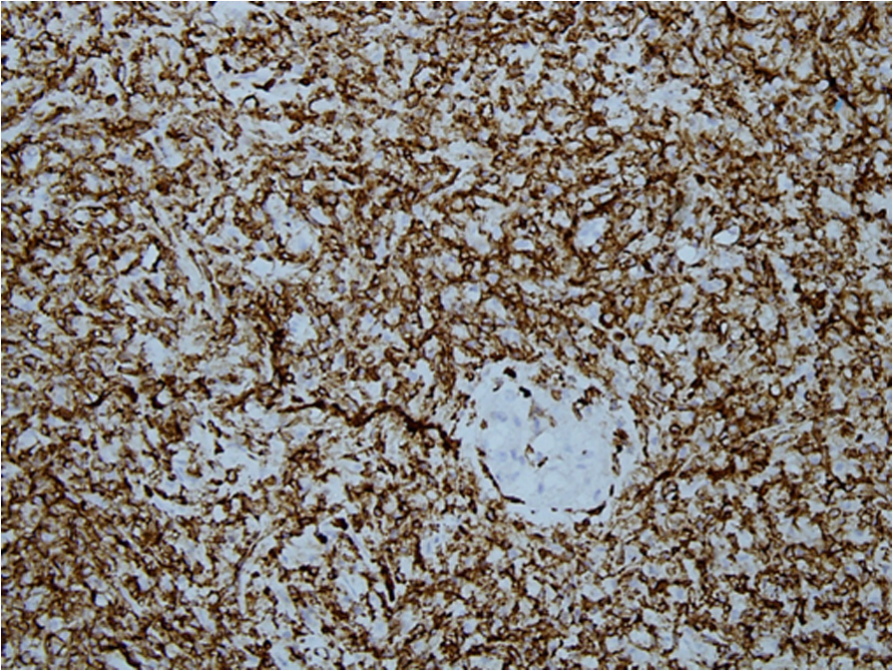
Immunohistochemical stain CD-3 positivity in the tumor cells.

**Figure 3:**
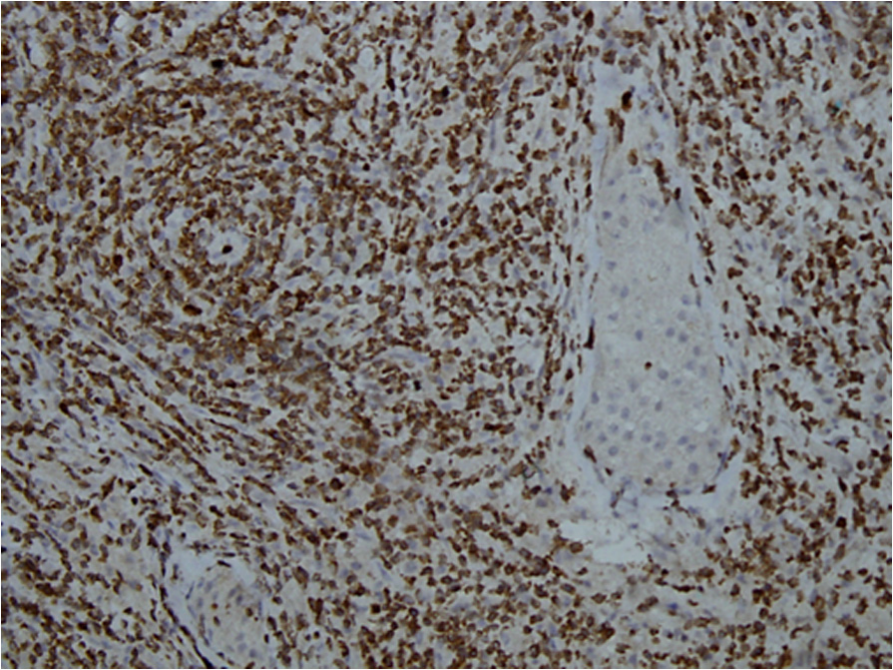
CD-43 immunostain was also positive in the neoplastic cells.

**Figure 4:**
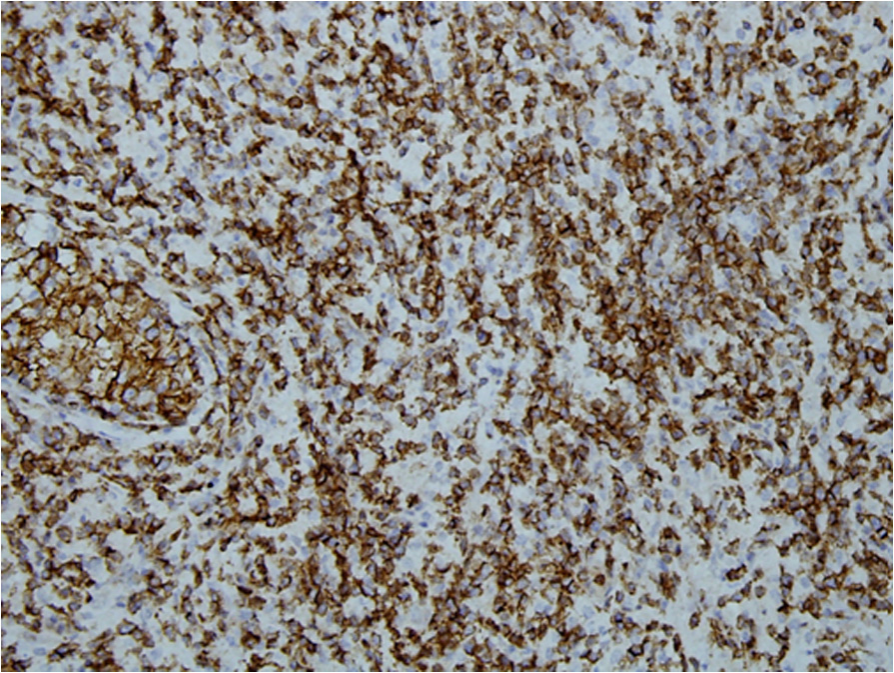
Strong expression of immunohistochemical stain CD-56.

Immunohistochemical stains were applied and neoplastic cells showed positivity with T-cell markers CD3 and CD 43 (Figures 2 and 3). CD 56 was also positive (Figure 4). Pan B (CD20), CD 4, CD 8, CD 5 and the epithelial marker cytokeratin were negative. Epstein-Barr virus (EBV) detection was done through immunohistochemistry by using EBV-latent membrane protein (LMP), which was also negative. The study was not experimental and no identifiable material was used in the manuscript, so this study did not require ethical approval.

## Discussion

Although primary testicular lymphoma (PTL) is the most common form of testicular cancer in men over the age of 60, and accounts for 7% of all testicular tumors, it accounts for only 1% of all non-Hodgkin’s Lymphomas and represents up to 38% of bilateral tumors. It is the most common testicular tumor in the patient age range 60 to 80 years and has a mean age on presentation of 64 years, but our patient unusually presented at 40 years of age [1-4].

In primary T-cell lymphomas, most case reports are of T-cell/Natural Killer (NK) cell lymphoma with very few cases of peripheral T-cell lymphoma (NOS). Our case represents one of those few cases of peripheral T-cell lymphomas previously reported in the literature [4-6]. EBV-associated T-cell/NK cell lymphomas most commonly involve the nasal cavities, and these are aggressive extranodal lymphomas. These are rarely encountered at sites other than the upper aero-digestive tract [6,7].

Clinical presentation is usually of a painless, unilateral testicular swelling. Symptoms of occasional sharp pain have been documented, with cases of abdominal pain with ascites being reported due to large retroperitoneal lymph nodes. Our patient had also had a painless testicular mass for the past three months. No pain or ascites was reported at the time of presentation [7,8]. Associated B symptoms (fever, night sweats, weight loss) usually present only in advanced stages, accounting for 25% to 41% of patients at diagnosis, and these were also not present in our patient [7-9].

A malignant lymphoma in which the tumor mass is limited to the testis at the time of clinical onset of the disease is rare. Since the first report of non-Hodgkin lymphoma manifesting as a testicular mass, described by Malassez in 1877, primary testicular lymphoma has attracted attention because of its rarity and poor prognosis. Testicular lymphoma is a lethal disease with a median survival of approximately 12 to 24 months [2,4,8]. Our patient had no other lymphadenopathy or splenohepatomegaly as was demonstrated by CT. Until now, 11-month follow up has been done and the patient is alive. He received chemotherapy, the conventional cyclophosphamide, adriamycin, vincristine, and prednisolone (CHOP) regimen initially. However, recurrence of the mass developed after 8 months and ipisilateral inguinal lymph node involvement was also present. The patient is now under radiotherapy treatment [7,8].

Most of patients with testicular large-cell lymphoma have a poor outcome and the long-term survival curves show no clear evidence of a substantial proportion of cured patients. Even for patients with stage I disease and good-risk International prognostic index (IPI), the outcome seems worse than that reported for diffuse large cell lymphoma (DLCL) at other sites [7-9]. There are reports of a high rate of extranodal recurrence and the frequent involvement of unusual sites. The duration of survival after relapse seems to be poor. Failures usually occur within 1 to 3 years after the initial therapy [7,9,10]. However, several late relapses of testicular lymphomas have been observed up to 14 years after diagnosis, especially in the central nervous system (CNS) and the contralateral testis, the latter raising the problem of distinguishing a new primary disease versus a late recurrence. The possibility of late CNS relapses has been reported in other studies as well, and is in marked contrast with the median time to CNS relapse of less than 1 year in patients with aggressive nodal lymphomas, so long-term follow up is mandatory for these patients [10,11].

This report indicates that testicular T-cell lymphoma deserves to be distinguished from the other testicular lymphomas for example, diffuse large B-cell lymphoma and Hodgkin’s lymphoma, because of the different treatment options. In fact, this lymphoma tends to occur at a younger age, to disseminate early, to have an aggressive course, and is strongly associated with EBV [4,10]. Response to multi-agent chemotherapy (such as CHOP, bleomycin, adriamycin, cyclophosphamide, vincristine, and prednisone (BACOP), or ProMACE-CytaBOM is often poor, even if complete remission is obtained, relapse may develop soon after. More aggressive treatment should be sought for this particular malignancy. A common protocol has still not been developed for refractory or relapsed peripheral T-cell lymphoma. Some commonly used regimens in these cases are ifosfamide, carboplatin, and etoposide (ICE), dexamethasone, high dose Ara C, and cisplatin (DHAP) and etoposide, methylprednisolone, cytarabine, and cisplatin (ESHAP). Also various clinical trials are underway using histone deacetylase inhibitors, proteosome inhibitors, nucleoside analogues, monoclonal antibodies and immunomodulatory drugs [10-12].

## Conclusions

Testicular peripheral T-cell lymphomas are rare and highly aggressive cancers, with clinical and histological differentials of seminoma and non-neoplastic conditions. Correct diagnosis with immunohistochemical techniques is mandatory for the proper treatment and further directed management of these tumors.

## Consent

Written informed consent was obtained from the patient for publication of this report and any accompanying images.

## Abbreviations

BACOP: Bleomycin adriamycin cyclophosphamide, vincristine, and prednisone; CNS: Central nervous system; CT: Computerized tomography; CHOP: Cyclophosphamide adriamycin vincristine, and prednisolone; DHAP: Dexamethasone high dose Ara C and cisplatin; DLCL: Diffuse large cell lymphoma; EBV: Epstein-Barr virus; ESHAP: Etoposide methylprednisolone cytarabine, and cisplatin; ESR: Erythrocyte sedimentation rate; ICE: Ifosfamide carboplatin and etoposide; LMP: Latent membrane protein; PTL: Primary testicular lymphoma; NK: Natural killer; IPI: International prognostic index

## Competing interests

The authors declare that they have no competing interests.

## Authors’ contributions

SH carried out the literature search and contributed in writing the manuscript. AA reviewed the slides and drafted the manuscript. All authors read and approved the final manuscript.
